# Why are the public so positive about colorectal cancer screening?

**DOI:** 10.1186/s12889-018-6106-1

**Published:** 2018-10-30

**Authors:** Linda N. Douma, Ellen Uiters, Danielle R. M. Timmermans

**Affiliations:** 1 Department of Public and Occupational Health, Amsterdam Public Health research institute, VU University Medical Center, Van der Boechorststraat 7, Amsterdam, 1081 BT The Netherlands; 2 National Institute for Public Health and the Environment (RIVM), Postbus 1, Bilthoven, 3720 BA The Netherlands

**Keywords:** Colorectal cancer, Screening, Public, Opinion, Perception

## Abstract

**Background:**

Colorectal cancer (CRC) screening is widely recommended. Earlier research showed that the general public are positive about CRC screening, as too the eligible CRC screening population. Among the eligible CRC screening population this positive perception has been shown to be associated with their perceptions of cancer, preventive health screening and their own health. It is unclear whether these concepts are also associated with the positive perception of the general public. Knowing this can provide insight into the context in which public perception concerning CRC screening is established. The aim of our study was to examine which main perceptions are associated with the public perception concerning CRC screening.

**Methods:**

An online survey was carried out in a Dutch population sample (adults 18+) among 1679 respondents (response rate was 56%). We assessed the public’s perceptions concerning cancer, preventive health screening, own health, and the government, and examined their possible association with public opinion concerning CRC screening.

**Results:**

The public’s positive attitude towards CRC screening is associated with the public’s positive attitude towards preventive health screening in general, their perceived seriousness of cancer, their belief of health being important, and their trust in the government regarding national screening programmes.

**Conclusion:**

Trust in the government and perceptions regarding the seriousness of cancer, preventive health screening and the importance of one’s health seem to be important factors influencing how the public view CRC screening. The public are likely to process information about CRC screening in such a way that it confirms their existing beliefs of cancer being serious and preventive screening being positive. This makes it likely that they will notice information about the possible benefits of CRC screening more than information about its possible downsides, which would also contribute to the positive perception of CRC screening.

**Electronic supplementary material:**

The online version of this article (10.1186/s12889-018-6106-1) contains supplementary material, which is available to authorized users.

## Background

Colorectal cancer (CRC) is one of the most common causes of cancer death in developed countries [1, 2]. Population-based CRC screening can reduce the incidence and mortality of CRC [3–6], which is why it is widely recommended [7–9]. Since January 2014, a national CRC screening programme exists in the Netherlands, provided by the government. The participation rate for this programme is relatively high (73%) [10], suggesting a positive perception of CRC screening among the eligible screening population, adults aged 55–75. Even though participation rates in other countries are typically lower, a generally positive perception of CRC screening among the eligible screening population is also found in other countries [11–17], with most people believing that CRC screening saves lives [15, 16] and that it is a good thing to do [13–16]. In addition to potential benefits, CRC screening also involves potential harms and risks (such as overdiagnosis, false negatives, false positives and risks associated with colonoscopy) [9, 18–21]. People seem to be more aware of the possible benefits of CRC screening than of its possible downsides [13, 14, 22]. Assessing whether the possible benefits for an individual outweigh the potential harms involves complicated information as well as personal values [23–25], making it a complex question to address. Therefore, making a well-informed and personal decision concerning CRC screening participation, with people truly understanding the possible benefits and harms as well as their own preferences, is increasingly seen as being important [23–27].

An individual’s personal perception and, consequently, personal decision concerning CRC screening might be affected by public opinion regarding CRC screening [28–31]. Therefore, it seems relevant to examine this public opinion. A common definition of public opinion is that it concerns the dominant opinion or the opinion of the majority on a topic relevant for the public [15, 16, 29, 32]. Previous research into public opinion has mostly been conducted in the field of sociology or political science, typically assessing public opinion by examining a large group of individuals representative for the public and determining their level of support [33–35] and/or attitude towards a certain issue or action [15, 16, 32–35]. When assessing public opinion by focussing on attitude, some studies made a distinction between personal attitude, which concerns whether someone thinks that CRC screening is a good idea for themselves, and collective attitude, which concerns whether someone thinks that CRC screening is a good idea for the Dutch population as a whole [29, 32]. Both personal and collective attitude affect the ‘overall’ attitude towards the CRC screening programme.

The majority of studies into CRC screening focused on examining perceptions of the eligible screening population within the direct context of individual participation [11–17], and not the perception of the general public, which includes people both inside and outside the eligible screening population [17, 36]. Earlier research showed that the Dutch public (adults 18+) are supportive of and positive about the CRC screening programme in the Netherlands, and were more aware of, and knowledgeable about, the possible benefits of CRC screening than about its possible harms and risks [37]. This is consistent with findings among the eligible screening population [17, 35, 38]. The lack of awareness of the possible harms and risks of CRC screening may, in part, explain why the public are positive about the CRC screening programme. In addition, the perception of CRC screening could also be affected by the perceptions regarding more general concepts related to CRC screening [30, 34, 39–42], as has been found in studies among the eligible screening population. The main general concepts examined in these studies were cancer [12, 15, 17, 43, 44], preventive health/cancer screening [15, 17, 43, 45], and one’s health [12, 43, 46]. These concepts seem to pertain to the core notion of what CRC screening entails (i.e. preventive screening to avoid becoming ill or to detect cancer in an early stage [7, 9]). Generally, people are more positive about CRC screening when they perceive cancer as being more serious [38, 44], see themselves as more susceptible to getting cancer [38], are more positive about preventive health screening [38, 45], and consider their health to be important [43, 46, 47]. Mixed results are found concerning the association with anxiety or worry about getting cancer [38, 44] and self-reported health status [38, 47]. Furthermore, the public’s perception of the government also seems relevant as the Dutch CRC screening programme is provided by the government [25]. People have a more positive perception towards facilities provided by the government when they trust the government or believe that the government has a responsibility in the public health domain [48, 49]. It is likely that people have had at least some previous experience with, or knowledge about, these more general concepts, resulting in pre-existing perceptions about them. These pre-existing perceptions can provide people with a mental framework, which is then used as a ‘short-cut’ to help understand and evaluate the less familiar subject of CRC screening by guiding which information is used and how to interpret it [30, 34, 40, 41, 50]. Although this ‘short-cut’ is often useful, it could also possibly affect how well-informed public opinion concerning CRC screening is, because information fitting well with pre-existing perceptions is generally noticed more, seen to be of more value, and remembered better [30, 34, 39, 40, 50, 51].

Our study aims to answer the following research questions:

1. What are the public’s perceptions concerning cancer, preventive health screening, own health, and the government?
2. Are the public’s attitude and level of support concerning the CRC screening programme associated with the public’s perceptions concerning cancer, preventive health screening, own health, and the government?

## Methods

### Questionnaire and participants

We recruited participants via a national online research panel (Flycatcher Internet Research, www.flycatcher.eu; ISO 26362). Members of this panel sign up voluntarily to participate in online research. The questionnaire was pre-tested among 36 members of the online panel; they were asked to comment on its comprehensibility, difficulty, length and intrusiveness. After the pre-test, some adjustments in wording and format were made. For our survey, 3000 panel members from the age of 18, diverse in education (low, intermediate, and high according to the International Standard Classification of Education (ISCED), 2011) and region were invited in December 2014 via e-mail to complete our online questionnaire.

### Measures (Fig. 1)

#### Public opinion concerning the CRC screening programme

##### Level of support

We assessed participants’ support for the CRC screening programme by asking whether they thought it was good that the programme existed in the Netherlands (5-point scale: 1 = *totally not good*, 5 = *totally good*) [33, 52].

**Fig. 1:**
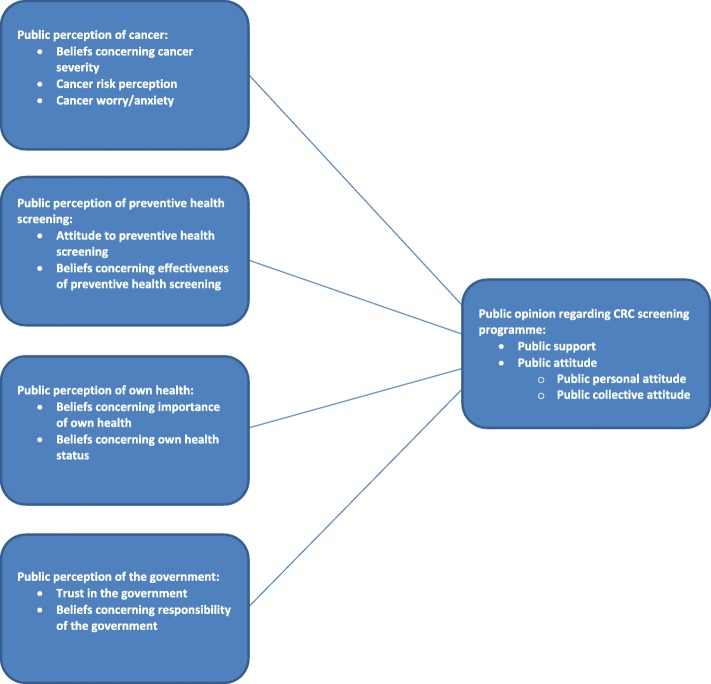
Measures used to assess public perceptions of cancer, preventive health screening, own health, the government, and the CRC screening programme

##### Personal and collective attitude

We first assessed participants’ collective attitude by asking them to evaluate the CRC screening programme with the Dutch population in mind (‘I believe the CRC screening programme to be … for the Dutch population’). Subsequently, we assessed participants’ personal attitude by asking them what they thought of the CRC screening programme for themselves (‘I believe participating in the CRC screening programme to be … for myself’). Regarding both attitude assessments, we asked participants to evaluate the CRC screening programme on six dimensions using 5-point semantic differential scales (bad/good; disturbing/reassuring; not meaningful/meaningful; not self-evident/self-evident; not frightening/frightening; unimportant/important). These dimensions were derived from the 10-item attitude scale used by Van Dam et al. [53].

#### Public perception of cancer

##### Beliefs concerning cancer severity

We assessed participants’ perception of the severity of cancer by asking to what extent they agreed with the following statements: 1. Cancer is very serious; 2. Cancer has major consequences for your life; 3. Cancer is very treatable; 4. Cancer means the end of your life; 5. Cancer is (virtually) impossible to prevent; 6. Cancer is more serious than other illnesses (5-point scales: 1 = *totally disagree,* 5 = *totally agree*) [54, 55].

##### Cancer risk perception

To assess participants’ risk perception concerning cancer, we asked what they thought their chance was of getting cancer in general (5-point scale: 1 = *very small,* 5 = *very big*), and how they perceived this chance compared to others (5-point scale: 1 = *much smaller*, 2 = *smaller*, 3 = *the same*, 4 = *greater*, 5 = *much greater*) [56, 57].

##### Cancer worry/anxiety

To assess participants’ worry and anxiety concerning cancer, we asked to what extent they worry about getting cancer, and to what extent the thought of getting it makes them feel anxious (5-point scales: 1 = *not at all,* 5 = *a lot*) [58, 59].

#### Public perception of preventive health screening

##### Attitude towards preventive health screening

We assessed participants’ attitude towards preventive health screening in general (such as cancer screening, screening for high blood pressure, screening for high cholesterol, etc.) by asking them to evaluate preventive health screening on six dimensions using 5-point semantic differential scales (bad/good; disturbing/reassuring; not meaningful/meaningful; not self-evident/self-evident; not frightening/frightening; unimportant/important) [53].

##### Beliefs concerning effectiveness of preventive health screening

We asked participants whether they thought that by regularly having their health examined they could detect health problems at an early stage (5-point scale: 1 = *totally disagree,* 5 = *totally agree*) [55]*.*

#### Public perception of own health

##### Beliefs concerning importance of own health

We asked participants to what extent they agreed with the statement ‘My health is very important to me’ (5-point scale: 1 = *totally disagree,* 5 = *totally agree*) [55].

##### Beliefs concerning own health status

We asked participants to report how they perceived their current health status to be (5-point scale: 1 = *poor*, 2 = *fair*, 3 = *good*, 4 = *very good*, 5 = *excellent*) [60].

#### Public perception of the government

##### Trust in government

First, we assessed participants’ trust in the government’s current policy in general on protecting and promoting people’s health (on a scale from one to ten: 1 = *none at all*, 10 = *a lot*) [61]. Second, we assessed participants’ trust in the government regarding national screening programmes by asking them to what extent they agreed with the following statements (following the format of Siegrist and Cvetokovich) [49]: 1. The government has people’s health as a priority when offering a national screening programme; 2. The government carefully considers the pros, cons and costs of a national screening programme; 3. The government communicates openly and fully about the pros, cons and costs of a national screening programme (5-point scales: 1 = *totally disagree,* 5 = *totally agree*).

##### Beliefs concerning responsibility of government

We asked participants what responsibility the government should have when it comes to people’s health, by asking them whether they agreed (yes/no) with the following statements: 1. The government has no responsibility when it comes to people’s health; 2. The government has the responsibility to provide public education concerning how people can stay as healthy as possible; 3. The government has the responsibility to provide national screening programmes (on a voluntary basis); 4. The government has the responsibility to ensure that people participate in national screening programmes.

### Statistical analysis

Missing data were very limited (with a maximum of 20 cases per variable) and were dealt with by performing mean imputation. Composite scores and scale scores (the means of the sum scores) were calculated when reliability was sufficient, based on factor analysis and the correlation between items or Cronbach’s alpha (α > .60).

We performed descriptive statistics for the public’s perceptions concerning cancer, preventive health screening, own health, and the government (see Additional file 1: Appendix A for the descriptive statistics of all initial single items used to measure these perceptions). Additionally, for descriptive purposes, scores for all variables were categorised, with scores of 1 and 2 classified as low or negative, scores of 3 as not high/not low or neutral, and scores of 4 and 5 as high or positive. For the variable ‘trust in government regarding protection and promotion of people’s health’ scoring 6 or higher was seen as having reasonable trust [61].

We conducted multiple linear regression analysis to determine the possible association between public opinion regarding the CRC screening programme and public perceptions concerning the main general concepts related to CRC screening. All analyses are based on the original range of scores and not on the categories we assigned. We explored three models, using three conceptualisations of public opinion (the dependent variable): 1. Level of support (model 1); 2. Personal attitude (model 2); and 3. Collective attitude (model 3). Variables regarding the public perceptions concerning cancer, preventive health screening, own health and the government were entered as independent variables. Backward selection was applied, where first all independent variables are entered in the model and the variable with the highest non-significant *p*-value (*p* ≥ .05) is then removed. Variables with significant effects are left in the final model. All analyses were carried out using IBM Statistical Package for the Social Sciences (SPSS) Software Version 22.0.

## Results

### Sample characteristics

The response rate to our survey was 56% (1679 participants). The sample characteristics are summarized in Table 1. A full description can be found in a previous publication [37]. Based on data from Statistics Netherlands [62], the distribution of the sample regarding gender, age, education and geographic location was representative of the Dutch population (i.e. the general public). Non-response analysis showed that people were more likely to have participated in our survey when older, higher educated or male.

**Table 1:** Demographic characteristics of the sample population

	Number (Percent)
Total	1679 (100)
Gender	
Men	903 (54)
Women	776 (46)
Age category	
18–24	123 (7)
25–34	235 (14)
35–44	258 (15)
45–54	362 (22)
55–64	285 (17)
65+	416 (25)
Education	
Low	544 (32)
Intermediate	681 (41)
High	454 (27)

### Public opinion regarding the CRC screening programme

People reported support for the CRC screening programme (*M* = 4.12, *SD* = .69) and to have a positive personal and collective attitude towards it (*M* = 4.06, *SD* = .76 and *M* = 4.07, *SD* = .60, respectively; Table 2).

**Table 2:** Public opinion regarding CRC screening and public perceptions of cancer, preventive health screening, own health, and the government (descriptive statistics)

Variables	M (SD)^1,a^	N (%)
Public opinion regarding CRC screening programme		
Level of support for CRC screening programme	4.12 (.69)	–
Attitude to CRC screening programme		
• Personal attitude	4.06 (.76)	–
• Collective attitude	4.07 (.60)	–
Public perception of cancer		
Beliefs concerning cancer severity	4.56 (.52)	–
Cancer risk perception	3.15 (.52)	–
Cancer worry/anxiety	2.86 (.76)	–
Public perception of preventive health screening		
Attitude to preventive health screening	3.89 (.81)	–
Beliefs concerning effectiveness of preventive health screening	3.56 (.84)	–
Public perception of own health		
Beliefs concerning importance of own health	4.34 (.60)	–
Beliefs concerning own health status	3.14 (.85)	–
Public perception of the government		
Trust in government regarding protection and promotion of people’s health	6.24 (1.78)	–
Trust in government regarding national screening programmes	3.34 (.69)	–
Beliefs concerning responsibility of government		
• Government has responsibility concerning people’s health (yes)	–	1321 (79)

^1^*N* = 1679

^a^Scores range from 1 (low/negative) to 5 (high/positive), except for the variable ‘trust in government regarding protection and promotion of people’s health’, where scores range from 1 (none) to 10 (a lot)

### Public perceptions of cancer, preventive health screening, own health, and the government

Based on reliability analysis, not all composite and scale scores as described in the Method section could be used as intended. Therefore, the items ‘cancer is very serious’ and ‘cancer has major consequences for your life’ were combined as a measure for ‘beliefs concerning cancer severity’. Additionally, the single item that asks about whether the government has a responsibility when it comes to people’s health is used as a measure for ‘beliefs concerning responsibility of the government’.

People believed cancer to be very serious (Table 2), but perceived the chance of getting cancer as neither small nor large and did not worry much about getting it. They had a positive attitude towards preventive health screening and found their health to be important. There is moderate trust in the government’s current policy on protecting and promoting people’s health as well as in national screening programmes specifically. A majority of our study sample sees a responsibility for the government when it comes to people’s health. More than half sees a responsibility in ensuring that people participate in national screening programmes.

### Association between public opinion regarding CRC screening and public perceptions of cancer, preventive health screening, own health, and the government

Table 3 shows the results for each of the three final models we analysed, with level of support (model 1), personal attitude (model 2) and collective attitude (model 3) as the three conceptualisations we used of public opinion. Level of support, personal attitude and collective attitude all show a positive association with beliefs related to the perception of cancer as well as to the perception of preventive health screening, own health, and the government. Personal attitude and collective attitude were associated with the same four beliefs: 1. Beliefs concerning cancer severity; 2. Attitude towards preventive health screening; 3. Beliefs concerning the importance of own health; and 4. Trust in government regarding national screening programmes. Level of support was associated with these same four beliefs as well as three additional ones: cancer risk perception, cancer worry/anxiety, and beliefs concerning the effectiveness of preventive health screening. The three final models differ slightly in explained variation, with the model with personal attitude as measure of public opinion having the largest explained variation (76%).

**Table 3:** Multiple linear regression models ^a^ of public opinion regarding CRC screening (level of support, personal attitude, collective attitude) and public beliefs regarding cancer, preventive health screening, own health, and the government ^b^

Model 1: Level of support as dependent variable (R^2^ = .62)		
Independent variable	B	95% CI ^b^
Beliefs concerning cancer severity	.145	.092–.198
Cancer risk perception	.058	.004–.111
Cancer worry/anxiety	.067	.029–.105
Attitude to preventive health screening	.315	.277–.353
Beliefs concerning effectiveness of preventive health screening	.074	.038–.110
Beliefs concerning importance of own health	.169	.121–.217
Trust in government regarding national screening programmes	.146	.107–.185
Model 2: Personal attitude as dependent variable (R^2^ = .76)		
Independent variable	B	95% CI
Beliefs concerning cancer severity	.061	.014–.109
Attitude to preventive health screening	.641	.609–.673
Beliefs concerning importance of own health	.090	.046–.133
Trust in government regarding national screening programmes	.106	.070–.141
Model 3: Collective attitude as dependent variable (R^2^ = .71)		
Independent variable	B	95% CI
Beliefs concerning cancer severity	.085	.044–.126
Attitude to preventive health screening	.454	.426–.481
Beliefs concerning importance of own health	.075	.038–.113
Trust in government regarding national screening programmes	.117	.086–.147

^a^Gender, age and education were not confounders in either model; uncorrected scores are shown

^b^Scores for all variables range from 1 (low/negative) to 5 (high/positive)

^c^Significant at *p* < .05

## Discussion

The Dutch public are generally supportive of, and positive about, the CRC screening programme. Their positive attitude towards preventive health screening in general, their perceived seriousness of cancer, their belief of health being important, and their trust in the government regarding national screening programmes are all associated with this positive attitude towards CRC screening. Additionally, the general public see a role for the government in promoting and protecting the public’s health.

Our findings are consistent with earlier research among the eligible CRC screening population into individual perceptions concerning concepts related to CRC screening [12, 15, 17, 43, 48]. This indicates that the opinion concerning CRC screening of people both inside and outside the eligible screening population is associated with a similar set of beliefs. To our knowledge, however, previous studies did not examine the relationship between the perception of CRC screening and the perceptions concerning cancer, preventive health screening, own health and the government all in one study. The set of beliefs that people have concerning a certain topic can provide them with a mental framework that is used as a ‘short-cut’ in forming an opinion about this topic by guiding the use and interpretation of information [30, 34, 40, 41, 50, 51]. This ‘short-cut’ is often very useful in daily life for quickly assessing how one thinks and feels about something, such as CRC screening. However, it could also lead to the public not noticing or remembering possibly relevant information because it does not fit well with their existing perceptions [30, 34, 39, 40, 51]. Following this rationale, regarding CRC screening, it could be that the public, given that they believe cancer to be serious and preventive health screening to be positive, are more likely to notice and remember information that confirms those beliefs, such as information about the possible benefits of CRC screening. Additionally, the public could be less likely to notice and remember information that does not confirm those beliefs, such as information about the possible downsides of CRC screening. They could also be more likely to interpret information about the possible downsides as not being downsides, or to discount their value, again confirming their existing belief that screening for cancer is something positive [39, 40, 51]. Thus, the set of beliefs that the public have concerning CRC screening could result in an overemphasis on the possible benefits of CRC screening and a lack of awareness and understanding of the possible downsides of CRC screening. This could affect the extent to which public opinion concerning CRC screening is well-informed [39, 40, 51]. Earlier research showed a lack of awareness of the possible harms and risks of CRC screening among the Dutch public [37]. The public being well-informed is of relevance because it is considered important that people make a well-informed personal decision concerning CRC screening [23, 26, 27] and public opinion might affect people’s personal view and decision concerning CRC screening [28–31]. Therefore, future research could focus on examining whether public perceptions regarding subjects related to CRC screening are associated with how well-informed the public are about CRC screening.

Based on previous research, we used three different measures of public opinion in our study: 1. Level of support; 2. Personal attitude; and 3. Collective attitude. When using level of support as a measure, we found three additional beliefs to be significantly associated with public opinion (cancer risk perception, cancer worry/anxiety, and beliefs concerning the effectiveness of preventive health screening) compared to when personal attitude or collective attitude was used as a measure. This difference might be because we assessed level of support using a single-item question, whereas we assessed personal and collective attitude using a six-item scale. In general, multi-item measures are considered to be more reliable and capable of capturing a broader meaning of a concept [63, 64], suggesting that personal attitude or collective attitude would be better measures to use. However, single-item measures could be more appealing for participants to answer, which could increase survey effectiveness [63], and they have been shown to sometimes have the same predictive validity as multi-item measures [63, 64]. In our study, using level of support as a measure does show a general similar result regarding public opinion to when personal or collective attitude was used as a measure. Further research is needed to examine the question of whether level of support, personal attitude or collective attitude would be best to use as a measure of public opinion. Additionally, we believe clearer definitions of conceptual terms such as ‘opinion’, ‘attitude’, ‘perception’ and ‘beliefs’ are also of importance, as these are often used interchangeably, making further theory development more complex.

Although it is fairly common practice to use a random sample of members of a national internet panel as participants, this might limit generalizability since people who participate in online research may differ in significant ways from people who do not participate in online research. Additionally, while we had a decent response rate of 56%, people were more likely to have participated in our online survey when older, higher educated or male. Furthermore, we are aware that the CRC screening uptake of 73% in the Netherlands [10] is relatively high compared to other countries, which might limit generalizability as well. However, although the participation rates in other countries are typically lower, a generally positive perception of CRC screening among the eligible screening population is also found in these other countries [11–17]. A strength of our study is that we examined the possible association between public opinion concerning CRC screening and public perceptions regarding various concepts related to CRC screening, providing a more comprehensive portrayal of the set of beliefs that may be relevant.

## Conclusion

Public opinion regarding the Dutch CRC screening programme is positive and supportive. Earlier research showed a lack of awareness of the possible harms and risks of CRC screening among the public [37], which may, in part, explain why the public are so positive. Additionally, our current study shows that the public’s trust in the government and their perceptions regarding the seriousness of cancer, preventive health screening and the importance of one’s health are important for how the public view CRC screening. When informing the public about CRC screening it would be useful to be aware of the fact that the public are positive about CRC screening, and that they are likely to process information about CRC screening in such a way that it confirms their existing beliefs of cancer being serious and preventive screening being positive [39, 40, 51]. This means that the public are likely to be inclined to notice and recall information about the possible benefits of CRC screening better than information about its possible downsides, which would thus contribute to a positive perception of CRC screening. Measures should be taken to ensure that the public are truly aware of both the possible benefits and the possible downsides of CRC screening in order to form a well-founded opinion.

## Additional file

Additional file 1: Appendix A. Descriptive statistics for all initial single items used to measure public opinion regarding CRC screening and public perceptions of cancer, preventive health screening, own health, and the government. (DOCX 20 kb)

## Abbreviations

CRC: Colorectal cancer screening
ISCED: International standard classification of education
ISO: International organisation for standardization
