# Supplementary material for: Why are the public so positive about colorectal cancer screening?
Source: BMC Public Health. 2018 Oct 30;18:1212. doi: 10.1186/s12889-018-6106-1 (PMC6208033; doi:10.1186/s12889-018-6106-1)
Supplement: Supplementary file 1 — Appendix A. Descriptive statistics for all initial single items used to measure public opinion regarding CRC screening and public perceptions of cancer, preventive health screening, own health, and the government. (DOCX 20 kb) [file 12889_2018_6106_MOESM1_ESM.docx]

**Additional file 1 for manuscript “Why are the public so positive about colorectal cancer screening?”**

***Appendix A:*** **Descriptive statistics for all initial single items used to measure public opinion regarding CRC screening and public perceptions of cancer, preventive health screening, own health, and the government**

| **Variables** | **M (SD)¹^,^ᵃ** | **N (%)** |
| --- | --- | --- |
| ***Public opinion regarding CRC screening programme*** |  |  |
| Level of support for CRC screening programme | 4.12 (.69) | - |
| Personal attitude to CRC screening programme   - I believe participating in the CRC screening programme to be *bad (1) – good (5)* for myself - I believe participating in the CRC screening programme to be *disturbing (1) – reassuring (5)* for myself - I believe participating in the CRC screening programme to be *not meaningful (1) – meaningful (5)* for myself - I believe participating in the CRC screening programme to be *not self-evident (1) – self-evident (5)* for myself - I believe participating in the CRC screening programme to be *not frightening (1) – frightening (5)* for myself - I believe participating in the CRC screening programme to be *unimportant (1) – important (5)* for myself   Collective attitude to CRC screening programme | 4.42 (.82)  4.20 (.92)  4.28 (.96)  3.75 (1.19)  3.50 (1.21)  4.24 (.91 | -  -  -  -  -  - |
| - I believe the CRC screening programme to be *bad (1) – good (5)* for the Dutch population - I believe the CRC screening programme to be *disturbing (1) – reassuring (5)* for the Dutch population - I believe the CRC screening programme to be *not meaningful (1) – meaningful (5)* for the Dutch population - I believe the CRC screening programme to be *not self-evident (1) – self-evident (5)* for the Dutch population - I believe the CRC screening programme to be *not frightening (1) – frightening (5)* for the Dutch population - I believe the CRC screening programme to be *unimportant (1) – important (5)* for the Dutch population | 4.61 (.66)  4.10 (.91)  4.40 (.78)  3.36 (1.09)  3.57 (1.09)  4.35 (.77) | -  -  -  -  -  - |
| ***Public perception of cancer*** |  |  |
| Beliefs concerning cancer severity |  |  |
| - Cancer is very serious | 4.53 (.58) | - |
| - Cancer has major consequences for your life | 4.59 (.54) | - |
| - Cancer is very treatable | 3.09 (.70) | - |
| - Cancer means the end of your life | 2.61 (.79) | - |
| - Cancer is (virtually) impossible to prevent | 3.18 (.84) | - |
| - Cancer is more serious than other illnesses | 3.44 (.89) | - |
| Cancer risk perception   - Perceived chance of getting cancer - Perceived chance of getting cancer compared to others | 3.28 (.66)  3.04 (.55) | -  - |
| Cancer worry/anxiety   - Worry about getting cancer - Anxious about getting | 2.79 (.80)  2.93 (.88) | -  - |
| ***Public perception of preventive health screening*** |  |  |
| Attitude to preventive health screening   - I believe preventive health screening to be *bad (1) – good (5)* - I believe preventive health screening to be *disturbing (1) – reassuring (5)* - I believe preventive health screening to be *not meaningful (1) – meaningful (5)* - I believe preventive health screening to be *not self-evident (1) – self-evident (5)* - I believe preventive health screening to be *not frightening (1) – frightening (5)* - I believe preventive health screening to be *unimportant (1) – important (5)* | 4.21 (.95)  4.11 (.97)  4.09 (1.04)  3.39 (1.23)  3.52 (1.17)  4.03 (.98) | -  -  -  -  -  - |
| Beliefs concerning effectiveness of preventive health  screening | 3.56 (.84) | - |
| ***Public perception of own health*** |  |  |
| Beliefs concerning importance of own health | 4.34 (.60) | - |
| Beliefs concerning own health status | 3.14 (.85) | - |
| ***Public perception of the government*** |  |  |
| Trust in government regarding protection and promotion of  people’s health | 6.24 (1.78) | - |
| Trust in government regarding national screening  programmes   - The government has people’s health as a priority when offering national screening programmes - The government carefully considers the pros, cons and costs of national screening programmes - The government communicates openly and fully about the pros, cons and costs of national screening programmes   Perception regarding responsibility of government   - Government has responsibility concerning people’s health (yes) - Government has responsibility to provide public education about staying healthy (yes) - Government has responsibility to provide national screening programmes (voluntary basis) (yes) - Government has responsibility to ensure participation in national screening programmes (yes) | 3.52 (.81)  3.47 (.82)  3.05 (.87)  -  -  -  - | -  -  -  1321 (79)  1541 (92)  1540 (92)  945 (56) |

¹ N = 1679

ᵃ Scores range from 1 (low/negative, or as labelled in the variable description) to 5 (high/positive, or as labelled in the variable description), except for the variable ‘trust in government regarding protection and promotion of people’s health’, where scores range from 1 (none) to 10 (a lot)
